# Metabolomic profiling of ^13^C-labelled cellulose digestion in a lower termite: insights into gut symbiont function

**DOI:** 10.1098/rspb.2014.0990

**Published:** 2014-08-22

**Authors:** Gaku Tokuda, Yuuri Tsuboi, Kumiko Kihara, Seikou Saitou, Sigeharu Moriya, Nathan Lo, Jun Kikuchi

**Affiliations:** 1 Tropical Biosphere Research Center, COMB, University of the Ryukyus, Nishihara, Okinawa 903-0213, Japan; 2 RIKEN Center for Sustainable Resource Science, Suehiro-cho, Tsurumi, Yokohama 230-0045, Japan; 3 RIKEN Antibiotics Laboratory, Suehiro-cho, Tsurumi, Yokohama 230-0045, Japan; 4 Department of Biological Sciences, Graduate School of Bioscience and Biotechnology, Tokyo Institute of Technology, Meguro, Tokyo 152-8550, Japan; 5 Graduate School of Medical Life Science, Yokohama City University, Suehiro-cho, Tsurumi, Yokohama 230-0045, Japan; 6 School of Biological Science, The University of Sydney, Sydney, New South Wales 2006, Australia; 7 Graduate School of Bioagricultural Sciences, Nagoya University, Furo-cho, Nagoya 464-8601, Japan

**Keywords:** termite, cellulose digestion, metabolomics, nuclear magnetic resonance

## Abstract

Termites consume an estimated 3–7 billion tonnes of lignocellulose annually, a role in nature which is unique for a single order of invertebrates. Their food is digested with the help of microbial symbionts, a relationship that has been recognized for 200 years and actively researched for at least a century. Although DNA- and RNA-based approaches have greatly refined the details of the process and the identities of the participants, the allocation of roles in space and time remains unclear. To resolve this issue, a pioneer study is reported using metabolomics to chart the *in situ* catabolism of ^13^C-cellulose fed to the dampwood species *Hodotermopsis sjostedti*. The results confirm that the secretion of endogenous cellulases by the host may be significant to the digestive process and indicate that a major contribution by hindgut bacteria is phosphorolysis of cellodextrins or cellobiose. This study provides evidence that essential amino acid acquisition by termites occurs following the lysis of microbial tissue obtained via proctodaeal trophallaxis.

## 1. Introduction

Termites consume an estimated 3–7 billion tonnes of lignocellulose annually, a highly significant process in tropical and subtropical ecosystems. Moreover, the digestion of cellulose (among other structural components of plant biomass) is 79–99% efficient, providing a direct link between termite feeding and the terrestrial carbon cycle, and a role for a single order of invertebrates that is unique in the biosphere [1,2]. It has long been recognized that termite cellulose digestion is assisted by gut-resident microbial symbionts; the mutualism has been actively researched for over a century, more recently in the context of modelling efficient large-scale and sustainable industrial processes of energy production from plant biomass [3].

Early breakthroughs in understanding termite digestion included the demonstration of anaerobic cellulose hydrolysis by intestinal protists (present in six of seven extant families, the so-called ‘lower termites’) [4], reductive H_2_/CO_2_ acetogenesis [5,6] and nitrogen fixation by gut bacteria [7,8]. In the past two decades, molecular methods have shown that termite hosts carry genes for endogenous cellulase, expressed either in the salivary glands or the midgut [9]. More recently, metagenomic studies have revealed the presence of highly diverse cellulase genes in the bacteria of termites, including those in which symbiotic protists are absent, the ‘higher termites’ [10]. The combination of endogenous cellulases with those of cellulolytic symbionts is thought to explain the high efficiency of the termite cellulose-digesting system [9,11], but major uncertainties remain. For example, although it is generally assumed that host-derived cellulases generate glucose that can be directly used by termites, to date glucose has not been identified as a direct end product, nor is the site of absorption of any metabolite accumulated from cellulose catabolism known [12,13]. Bacterial cellulases are coded genetically and expressed through RNA [10], but the degree of their contribution to the intestinal cellulolysis remains unclear. In addition, the fate of ingested lignin also remains unclear [3].

Strict nitrogen conservation in wood-feeding termites is made necessary by the low levels of this element in wood (0.05–1.4%) [14]. Symbiotic bacteria demonstrate the genetic capacity to fix atmospheric nitrogen and to synthesize essential amino acids without organic nitrogen precursors [15]; they can also degrade uric acid with acetate and ammonia as end products available for assimilation by other microbiota [8]. These processes may be the basis of an efficient nitrogen economy, but there is no direct evidence that essential amino acids (a form of nitrogen that could be assimilated by the host) are made available to termite tissues. A site of uptake has not been identified, although proctodeal trophallaxis provides a possible route.

Two-dimensional nuclear magnetic resonance (2D-NMR) is an emerging technology that offers the generation of comprehensive metabolic profiles from small samples containing mixtures of compounds, and in combination with isotopic labels allows the dynamics of complex catabolic and anabolic systems to be analysed over a time series at an atomic level [16,17]. For example, ^13^C-precursors have been used to map metabolic processes during insect morphogenesis [18] and to define agonistic interactions between resident gut bacteria and enteropathogens in mammals [19]. In the context of termite digestion, the technique may tie function to sequence, thus permitting the large number of metabolic events and interactions that occur after feeding to be placed in temporal order and located spatially within the intestine and other host organs. In this paper, experiments are described in which ^13^C-cellulose was fed to the dampwood termite *Hodotermopsis sjostedti*, and the ensuing labelled metabolites assayed and located *in situ*. Specialized ordination was employed to separate clusters of metabolites that were synchronized with cellulose hydrolysis from those that were not, leading to the conclusion that free essential amino acids were not released to the hindgut lumen, but acquired by another route, most probably the digestion of microbial tissue ingested via proctodeal trophallaxis.

## 2. Material and methods

Detailed information on materials and analytical methods is provided in the electronic supplementary material. In brief, *H. sjostedti* termites and logs were collected on Yakushima Island, Kagoshima Prefecture, Japan. Termites were maintained with logs and soil obtained from the collection site and stored at room temperature until use. Mature worker-caste termites were used for all experiments. Twenty-five termites were maintained with ^13^C-cellulose (synthesized by *Acetobacter xylinum*), in which the degree of ^13^C incorporation to the cellulose was determined with isotope-ratio mass spectrometry (IR-MS) to be approximately 93%. Twenty individuals were randomly sampled from the 25 stable isotope-labelled termite individuals. An equal numbers of termites (i.e. 20), prior to feeding ^13^C-cellulose, were dissected as controls. Following dissection to separate regions (electronic supplementary material, figure S1), the gut samples were immediately frozen on dry ice and freeze-dried. Further processing for metabolite extraction and NMR was as previously described [20–22]. Digitized NMR data were normalized using 1 mM sodium-2,2-dimethyl-2-silapentane-5-sulfonate (DSS) and intensities were estimated per microlitre of gut fluid, based on the following gut section volumes: foregut, 1.7 ± 0.2 µl; midgut, 2.0 ± 0.4 µl; anterior hindgut, 16.9 ± 2.4 µl; posterior hindgut, 6.9 ± 1.7 µl (*n* = 10; see the electronic supplementary material for further details on calculation of gut volume). NMR data were also subjected to hierarchical cluster analysis using R software (R Development Core Team, Vienna; http://www.R-project.org), and Kruskal's non-metric multidimensional scaling (NMDS) was applied to represent the dissimilarity of dependence between metabolites by Euclidean distance in a two-dimensional ordination. Significant dissimilarity between clusters of metabolites was assessed by a multiple response permutation procedure (MRPP) based on 9999 permutations [23].

## 3. Results

### (a) Profiles of metabolites derived from ^13^C-cellulose in the digestive system of *Hodotermopsis sjostedti*

Administration of ^13^C-cellulose resulted in a total of 256 signals detected in the digestive system with 2D heteronuclear single quantum coherence (HSQC)-NMR. Among these, 185 signals were assigned to 46 known metabolites (electronic supplementary material, figure S2 and table S1). Detailed distribution profiles of metabolites are provided in the electronic supplementary material. A comparison of 1D ^1^H and ^13^C NMR spectra derived from each of the four regions of the gut was also performed (electronic supplementary material, figure S3), and revealed major differences in the metabolite composition in each of these regions, including formate (observed only in the hindgut) and bicarbonate (observed from the midgut to the hindgut) in addition to metabolites detectable with 2D HSQC-NMR.

### (b) Time series of intestinal ^13^C-cellulose metabolism

**Figure 1.**
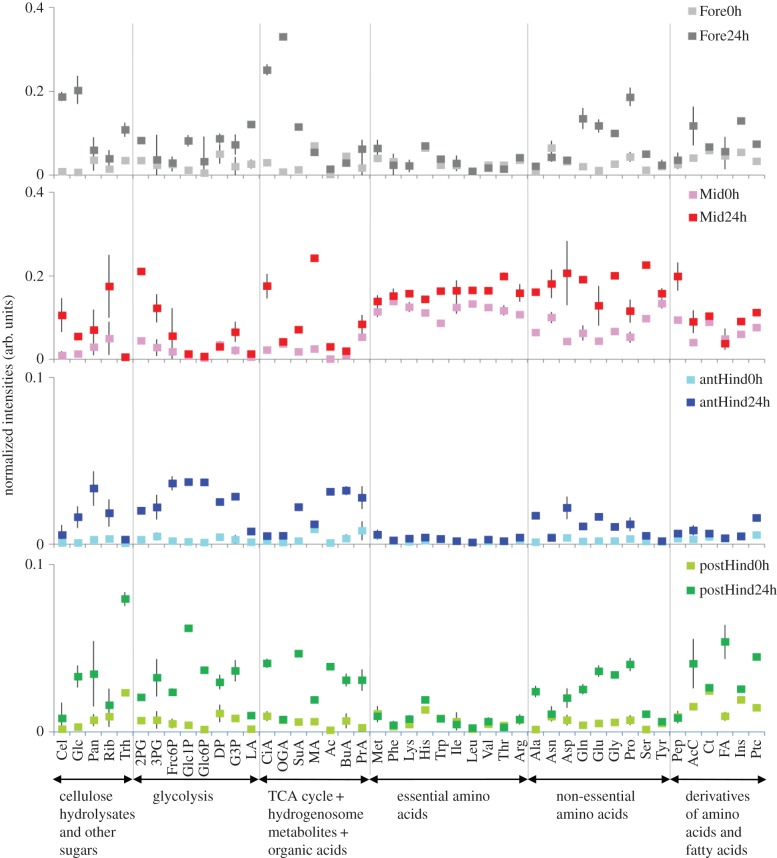
Comparisons of ^13^C-HSQC-NMR signal intensities of metabolites between 0 and 24 h post-feeding in sequential gut regions of *H. sjostedti*. Abbreviations of metabolites are according to electronic supplementary material, table S1. Normalized intensity values of metabolite signals (using DSS) per 1 µl of gut volume (represented as arb. units) are shown as means ± s.d. The termite gut regions are abbreviated as follows: Fore, foregut; Mid, midgut; antHind, anterior hindgut; postHind, posterior hindgut.

During ^13^C-cellulose feeding, overall ^13^C incorporation into intestinal metabolites steadily increased and ^13^C incorporation into the body was observed from around 24 h (electronic supplementary material, figure S4). Figure 1 shows a comparison of normalized intensities per microlitre of gut fluid between 0 h (i.e*.* immediately prior to feeding) and 24 h. In the foregut and midgut, some glycolytic pathway intermediates increased in intensity after 24 h, while in the anterior and the posterior hindguts, signals for all glycolytic metabolites increased markedly after 24 h. Signals for some TCA cycle metabolites such as citrate and 2-oxoglutarate (OGA, also known as α-ketoglutarate) were detected in the foregut and midgut. Evidence for the production of succinate (SuA), which can be produced either in the TCA cycle, during anaerobic fermentation by Bacteroidetes [24], or in anaerobic protist hydrogenosome (an organelle characteristic of parabasalian protists) metabolism, was found in the hindgut. SuA and volatile fatty acids (VFAs) such as acetate (Ac), butyrate (BuA) and propionate (PrA) were more abundant in the anterior hindgut than the posterior hindgut, while the converse was true for citrate (CiA) and OGA.

Signals from essential amino acids were strongest in the midgut for both zero time and 24 h samples, and relatively weak in other areas of the gut. Signals from non-essential amino acids were spread throughout the gut, being strongest in the midgut and hindgut.

**Figure 2.**
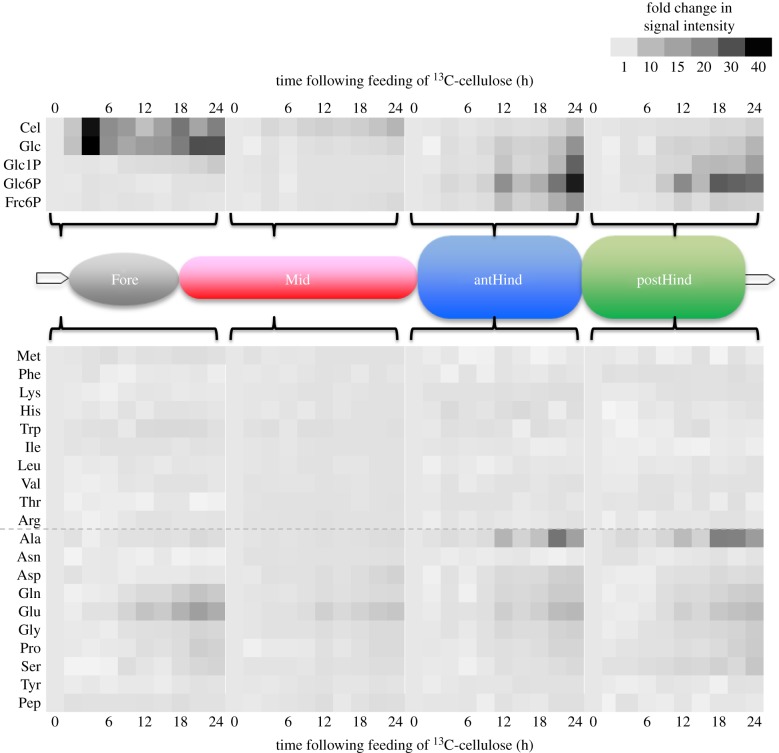
(*a*) Heat map indicating time-dependent fold changes of ^13^C-HSQC-NMR signal intensities of amino acids and metabolites associated with cellulose hydrolysis and the glycolytic pathway. Signal intensities at 0 h were defined as 1.0 in all gut regions. Abbreviations are according to the electronic supplementary material, table S1. The termite gut regions are abbreviated as follows: Fore, foregut; Mid, midgut; antHind, anterior hindgut; postHind, posterior hindgut.

To further investigate the incorporation of ^13^C into metabolites associated with cellulolytic hydrolysates and amino acids, signal intensities were measured at 1–3 h intervals following administration to termites. A heat map showing relative intensities of metabolites in different gut regions over this period is shown in figure 2. In the foregut, the signal intensities of cellobiose and glucose immediately increased and reached up to 50-fold that of the zero time reading after only 3 h of feeding. These signals in the foregut were detected throughout the experimental period (figure 2), with a 20-fold level maintained after 24 h. In the midgut, signals from cellobiose and glucose increased to a lesser degree, peaking at 11-fold and fourfold after 24 h. In the hindgut, signals corresponding to cellobiose and glucose began to intensify after approximately 12 h (figure 2), and remained at 8–18-fold levels in the anterior hindgut and 5–11-fold levels in the posterior hindgut until 24 h after administration of ^13^C-cellulose. Signals from the glycolytic pathway increased up to sevenfold in the foregut and threefold in the midgut, respectively (figure 2), while in the hindgut they reached up to 38-fold after 24 h.

Essential amino acid signals in the foregut increased up to approximately threefold by 18 h in some cases (Met, Trp), but generally did not increase greatly over the 24 h. The highest overall increases of essential amino acids were found in the midgut, with signals reaching around twofold by about 12 h and remaining at these levels until 24 h. In the hindgut, some essential amino acid signals (e.g. Phe, Lys, His) increased up to threefold while others remained low. Some essential amino acid signals (e.g. Met, Ile and Thr) slightly decreased after 24 h in the posterior hindgut. Signal intensities of non-essential amino acids were generally higher across all gut regions, exhibiting up to 30-fold increases. In the foregut, these increases mainly appeared after 9 h or later, while they commenced after 3 h in the midgut and 6–9 h in the hindgut (figure 2).

To elucidate the temporal relationship between cellulose degradation and the metabolites detected, an ordination of metabolites by NMDS was performed (see table 1; electronic supplementary material, figure S5). This was based on clustering analysis of the temporal dynamics of metabolite signal intensities over 24 h with average linkage inferred from Euclidean distance. NMDS of each gut segment suggested that temporal changes in signal intensity of cellulose hydrolysates in the foregut were not directly linked to those of other metabolites. However, temporal changes in signal intensities of cellulose hydrolysates in the midgut and hindgut were grouped (i.e. synchronized) with those of catabolites associated with the glycolytic pathway and either the TCA cycle (with the exception of OGA) or hydrogenosome function. The significance of these groupings was confirmed by an MRPP (*p* = 1 × 10^–4^ in all gut regions). Trehalose, lactate and VFAs occurred in the same group of cellulose catabolites in the anterior and posterior hindguts (table 1).

**Table 1.** Metabolites showing synchronomous temporal changes in signal intensities with those of cellulose hydrolysates based on NMDS.^a^ Abbreviations of metabolites are according to electronic supplementary material, table S1.

	foregut	midgut		anterior hindgut		posterior hindgut	
glycolysis	none	2PG		2PG	Glc1P	2PG	Glc1P
		3PG		3PG	Glc6P	3PG	Glc6P
		Frc6P		Frc6P	G3P	Frc6P	G3P
		G3P		LA	DP	LA	DP
TCA cycle + hydrogenosome	none	CiA		CiA		CiA	
		SuA		SuA		SuA	
VFAs		Ac		Ac	BuA	Ac	BuA
				PrA		PrA	
essential amino acids	none	none		Lys		Lys	
				Val			
non-essential amino acids	none	Ala	Glu	Ala	Gly	Ala	Gly
		Asn	Gly	Asp	Pro	Asp	Pro
		Asp	Pro	Gln	Ser	Gln	Ser
		Gln	Ser	Glu		Glu	
amino acid derivatives	none	AcC		AcC		AcC	
				Ptc		Ptc	
other sugars	none	Pan		Pan	Trh	Pan	Trh
		Rib		Rib		Rib	

^a^Provided as electronic supplementary material, figure S5.

Temporal dynamics in the intensity of glucose-1-phosphate (Glc1P) signals in the midgut were different from metabolites associated with cellulose catabolism, and instead formed a closely related cluster with glucose-6-phosphate (Glc6P) (electronic supplementary material, figure S5, blue circle). A statistical test showed that the Glc1P and Glc6P cluster and the cluster representing the products of cellulose dissimilation (electronic supplementary material, figure S5, dotted circle) in the midgut were significantly different (MRPP, *p* = 0.0039). By contrast, Glc1P signals in the hindgut were strongly associated with those of cellulose hydrolysates and glycolytic pathway intermediates (table 1; electronic supplementary material, figure S5). The production of glycerol-3-phosphate and acetate always occurred with cellulose catabolism in the midgut and hindgut, whereas trehalose, propionate and butyrate were produced in conjunction with metabolites involved in cellulose catabolism in the hindgut.

NMDS suggested that the temporal changes of all essential amino acid signals were not synchronized with those of the metabolites of cellulose dissimilation in the midgut. A similar pattern was found in the hindgut, with the exception of Lys and Val (table 1; electronic supplementary material, figure S5). In all gut regions, the signal intensities of most non-essential amino acids gradually increased to 30-fold of their original values during the 24 h experimental period, with the exceptions of Asn and Tyr, which tended to decrease (figure 2). NMDS showed that the temporal dynamics of non-essential amino acids were closely related to cellulose metabolism in the midgut, with the exception of Tyr. Neither Asn nor Tyr was grouped with metabolites of cellulose catabolism in the hindgut (table 1; electronic supplementary material, figure S5).

## 4. Discussion

**Figure 3.**
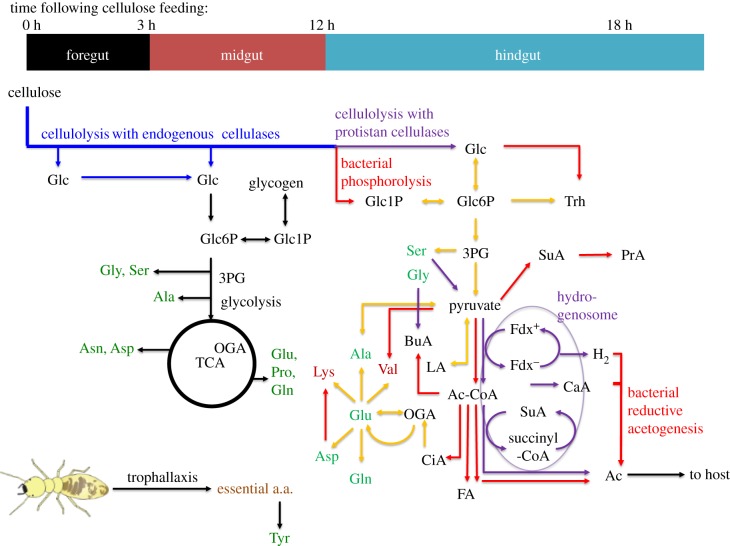
Time-dependent schematic describing cellulose catabolism in the digestive system of *H. sjostedti*. Cellulolysis via endogenous cellulases is depicted by blue arrows, whereas other pathways in the host termite are indicated by black arrows. Red arrows represent bacterial metabolic pathways in the hindgut, and purple arrows indicate protistan metabolic processes. Metabolic pathways assumed to be retained by both protists and bacteria are shown in yellow. The protistan and bacterial metabolic pathways were inferred from the published genomes of the parabasalian protist *Trichomonas vaginalis* [25], the rumen bacterium *Fibrobacter succinogenes* [26], the free-living symbiotic bacterium of termites *Treponema azotonutricium* (NCBI reference number NC_015577) [27] and the endosymbiont of termite protist Rs-D17 (*Candidatus* Endomicrobium trichonymphae) [28]. Essential amino acids are represented in brown and non-essential amino acids in green. Fdx, Ferredoxin. Abbreviations of other metabolites are according to the electronic supplementary material, table S1.

Based on the results presented here and in previous genomic, physiological and biochemical studies (see Introduction), a scheme for termite cellulose digestion and metabolism is proposed in figure 3. Details concerning the processes in different parts of the termite gut system are provided below.

### (a) Cellulose hydrolysis and carbon metabolism in the foregut and midgut

During feeding in lower termites, wood fragments are produced by the grinding action of the mandibles, and mixed with host cellulases produced in the salivary glands. This mixture then moves to the foregut, which consists of the oesophagus, crop and muscular proventriculus. Despite numerous studies on host-derived cellulases [3,9], the contribution of these enzymes to host metabolism is not well established. Efficient decomposition of crystalline cellulose into hydrolysates such as glucose and cellobiose in the hindgut of lower termites is generally considered to require the combined action of cellobiohydrolases and endoglucanases [3]. Since endogenous termite cellulases are only of the latter type, their ability to generate cellulose hydrolysates that are directly usable by the host has remained unclear. Although these enzymes have been shown to degrade crystalline cellulose to a minor extent in laboratory assays, to date there have been no demonstrations of *in situ* activity of these enzymes. In this study, termites were fed cellulose produced by *A. xylinum*, which is generally known to be highly crystalline and pure [29]. Although this substrate is distinct from trilaminate lignocellulose occurring in plants, this study provides the first firm evidence of *in situ* cellulolysis in the foregut and the midgut, as well as metabolism of the breakdown products (note that *H. sjostedti* expresses endogenous cellulases only in the salivary glands, and not the midgut [30]). Previous analyses with chromatography and zymograms indicated that most cellulases present in these gut compartments were distinct from those of the hindgut microbiota [31–33].

The ability of host endoglucanases to produce cellulose hydrolysates is probably enhanced by the mechanical grinding of wood fragments by the mandibles and proventriculus. According to a previous report using another lower termite, such grinding reduces wood to fragments less than 20 µm in length [34]. During this mechanical process, some crystalline cellulose within the fragments is likely to be converted into an amorphous state, allowing endoglucanases to act. The production of cellulose hydrolysates in the foregut did not display any significant correlation with the temporal dynamics of other metabolites detected. This indicates that the foregut is not a major site of catabolism of cellulose breakdown products. The epithelial cells of the foregut have a thick cuticle and lack absorptive morphological characteristics [35]. This makes the foregut an unlikely site of glucose absorption and metabolism. Instead, cellulose hydrolysates produced in the foregut probably pass through to the midgut (figure 3). Additional cellulose hydrolysates would be produced in the midgut by wood fragments and endogenous cellulases entering from the foregut (figures 2 and 3). Ordination of midgut metabolites by NMDS suggested that cellulose hydrolysates are metabolized by β-glucosidases and the glycolytic pathway and TCA cycle in this region. An exception to this pattern was the TCA cycle intermediate OGA. This can be explained by the fact that OGA is a key compound of several biochemical pathways and that both the production and consumption of OGA can be regulated by anaplerosis and cataplerosis [36]. Considering the synchronized production of Glu with cellulolysis, the TCA cycle might have been balanced by cataplerotic flux converting residual OGA (with ammonium) into Glu, a part of which was then further catalysed to other non-essential amino acids of the glutamate family such as Gln and Pro [37]. A second compound that did not group with cellulose hydrolysates in the midgut in the NMDS analysis was Glc6P (instead, Glc6P clustered with Glc1P and is probably an intermediate for glycogenesis in this tissue; figure 3). Although Glc6P is synthesized from Glc and flows into the glycolytic pathway, it was probably responsive to the regulation of glycogenesis. Finally, the presence of residential bacterial activity in the midgut cannot be excluded due to a weak but synchronized signal of acetate with cellulolysis.

### (b) Cellulose hydrolysis and carbon metabolism in the hindgut

Considerably increased signal intensities of cellulose hydrolysates and glycolytic pathway metabolites were found in the hindgut within 24 h of feeding. Taking the large hindgut volume into account (86% of total gut volume), this result confirms the primary role this gut segment plays in energy production from cellulose. In *H. sjostedti*, the hindgut is inhabited by 13 parabasalid and six oxymonad anaerobic protist species, all of which lack mitochondria [38], as well as numerous types of bacteria [39,40]. Termite protists are also known to contain intracellular and extracellular bacteria [15,41]. Recent studies suggest that these protists produce a variety of cellulases and hemicellulases [3,42], and that 85% of the acetate present in hindgut fluid is derived from cellulose [43]. In addition, a study indicated that a cultured protistan species from *Zootermopsis* sp. (from the same family as *H. sjostedti*) preferentially degraded cellulose as a fermentable energy source rather than other substrates such as xylan and chitin [44], suggesting the relative importance of cellulose as a substrate for the metabolic system in the hindgut.

Parabasalian protists are assumed to possess hydrogenosomes and produce acetate, H_2_ and CO_2_ [43–48]. Although this organelle has not been characterized from the hindgut fauna of termites, hydrogenase activity has been found in the hydrogenosomal fraction of the parabasalid protists of termites [46]. Oxymonads appear to lack both mitochondria and hydrogenosomes [41], and their energy metabolism is currently obscure. However, the presence of functions similar to hydrogenosomes in oxymonads is plausible [49]. This study observed synchronized production of VFAs with cellulolysis in the anterior and posterior hindgut, confirming that these compounds are among the end products of cellulose metabolism by the intestinal microbes (figure 3) [43]. A major proportion of acetate synthesis is likely associated with the activities of these protists, which occupy most areas of the hindgut lumen. Bacteria (especially spirochaetes) are also likely to have contributed to acetate production via reductive H_2_/CO_2_ acetogenesis [5,13,27,50]. The origins of other VFAs are currently obscure, but the bacterial community is most likely to be responsible.

The anterior hindgut showed the most remarkable increase in signal intensities of glycolytic metabolites compared with other gut positions (figure 1). The anterior hindgut occupies approximately 60% of the total gut volume (i.e. 16.9 µl of 27.6 µl), suggesting that glycolysis in the termite digestive system takes place primarily in this region. The anterior hindgut is also the most anoxic region of the gut in termites, although the periphery of this region is oxygenated [51]. The temporal dynamics of intermediate metabolites related to either bacterial fermentation (i.e. CiA, OGA and SuA) or protistan hydrogenosome metabolism (i.e. SuA) in the hindgut were synchronized with cellulose hydrolysate dynamics. This study demonstrated that SuA is an important intermediate in the cellulose metabolism of the anterior hindgut. The relative abundance of SuA was also reported from *Z. nevadensis* [52], suggesting that this compound may be abundant in termite hindguts, at least those in the family Termopsidae. It is currently not clear whether the strong intensity of SuA in this area of the gut was principally from protistan hydrogenosome metabolism [25] alone, or from a combination of protists and fermentation by anaerobic and aerotolerant bacteria such as Bacteroidales, lactococci and enterococci (some of which are localized in the periphery [39,40]; Bacteroidales are often present as ecto- and endosymbiotic associates of the protists [41]), but the synchronized synthesis of propionate suggests that SuA might be converted to propionate via the succinate–propionate pathway [53].

The posterior hindgut is likely to be less anaerobic than the anterior hindgut [51,54]. A previous study using another lower termite species showed that the number and density of anaerobic flagellates decreased from the anterior to the posterior hindgut. Most parabasalid flagellates were observed only in the anterior hindgut, though some oxymonadids were present in the posterior hindgut [55]. Metabolic processes occurring in the posterior hindgut are therefore more likely to be representative of oxymonadids and bacteria than parabasalids. This hypothesis is partially supported by dissimilarities in signals from metabolites such as CiA, OGA and SuA between the anterior and the posterior hindguts. Further studies, especially of the axial profiles of oxygen and the microbial community, are required to elucidate which microorganisms play a major metabolic role in the posterior hindgut.

### (c) Temporal dynamics of Glc1P shed light on the previously neglected contribution to lower termite cellulose metabolism by hindgut bacteria

The temporal dynamics of Glc1P in both regions of the hindgut were strongly synchronized with metabolites associated with cellulose hydrolysates. This indicates that Glc1P is produced as a result of phosphorolysis of cellodextrins or cellobiose as opposed to glycogenesis. As production of Glc1P during such phosphorolysis is not known in eukaryotic organisms (with the exception of some fungi), anaerobic bacteria are likely to be responsible [56]. Indeed, a large number of genes encoding cellodextrin or cellobiose phosphorylases have been found in metagenomic analyses of gut bacteria of higher termites, and several genes were identified in spirochaetes, notably *Treponema* spp. from the luminal fluid of the hindgut [10]. A major role for such bacterial enzymes in wood polysaccharide hydrolysis in higher termites was previously proposed [10]. Cellodextrin or cellobiose phosphorylases are thought to play an important role in catalysing oligosaccharides into simple sugars in the bacterial cytoplasm in the higher termites, but the role of bacteria in cellulose digestion in lower termites has been overshadowed by the presumed dominance of protists. Recent genome sequencing of the spirochaete *T. pirimitia* ZAS-2, from *Z. angusticollis* (a close relative of *H. sjostedti*), has demonstrated the presence of genes encoding both cellulase and cellobiose phosphorylase (NCBI reference no. NC_015578) [27], supporting earlier demonstrations of growth of spirochaetes from this species on cellobiose [57]. Moreover, some *Treponema* species are known as ectosymbionts of the protists [41]. These bacteria are able to adhere to wood fragments present in the luminal space of the hindgut. The demonstration of a strong association between the dynamics of Glc1P and cellulose hydrolysates provides support for the role of bacteria in cellulolysis and for cellobiose and cellodextrins being major organic substrates metabolized by gut bacteria in lower termites. Thus, the present results conflict with traditional views on the role of bacteria in cellulose fermentation in lower termites, and the pathways related to the cellulose-derived carbon involving bacterial hemoacetic fermentation. The recently proposed participation of *Treponema* in cellulolysis based on the metagenomic analysis of a higher termite [10] may also be applicable to *H. sjostedti,* and the relative roles of bacteria versus protists in polysaccharide fermentation in lower termites may need to be re-evaluated. The evolutionary shift from the protistan-dominant to the protistan-free gut systems of higher termites might have occurred with stepwise changes to the gut community and a reassortment of relative roles. This is more plausible than a ‘loss and replacement mechanism’.

### (d) Amino acid synthesis and acquisition

Hindgut bacteria in some termites are known to fix atmospheric nitrogen, and some species whose genomes have been sequenced contain the pathways for synthesizing essential amino acids using fixed nitrogen [15]. It is not known how much atmospheric nitrogen is fixed by the bacterial community in *H. sjostedti*; however, only limited amounts of N_2_ fixation are known to occur in members of the related genus *Zootermopsis* (0.05–0.33 μg of the nitrogen per gram fresh body weight) [58]. Diverse genes encoding proteins associated with N_2_ fixation (*nif*H) are nevertheless found in the guts of termites including *H. sjostedti, Z. angusticollis* and *Z. nevadensis* [59,60]. In addition, a mutualistic symbiont from the gut microbial community of *Z. nevadensis* has been demonstrated to be a N_2_-fixing diazotroph *in vitro* [59]. The route by which microbe-derived nitrogen is transferred to termite tissues has not been determined.

The present analyses showed that the midgut of *H. sjostedti* has higher normalized intensities of free essential amino acids than other gut regions, implying higher concentrations relative to the rest of the alimentary canal (figure 1). These results are in agreement with a previous study of a lower termite [61]. These amino acids appear to be derived from the digestion of microorganisms that are present in the proctodeal fluid that is transferred via nest-mates through trophallaxis [62,63]. Within 24 h, levels of ^13^C-derived essential amino acids in the midgut had increased markedly, ranging from a 9.5% increase in phenylalanine to a 90.3% increase in tryptophan (figure 1). Given that the termites had been fed exclusively on ^13^C-derived cellulose, this increase in the midgut can be attributed to the incorporation of ^13^C into essential amino acids by microbes in the hindgut, followed by transfer of these microbes to nest-mates by proctodeal trophallaxis and their eventual digestion. The digestion of these microbes could be mediated through salivary β-glucosidases, which attack β-1,3-glucans present in microbial cell walls [64,65], and proteinases, lysozymes and chitinases that are known to be produced in the midgut of *H. sjostedti* [66].

The present results show that cellulose hydrolysis starts in the posterior hindgut after 12 h (figure 2). This was not matched by an increase in essential amino acid signal intensity in the hindgut (figure 2), and even after 24 h, signals for essential amino acids in the hindgut were very low (figure 1). However, given the increase in ^13^C in essential amino acids in the midgut after 24 h (via proctodeal trophallaxis; see above), we can conclude that ^13^C must have been incorporated into essential amino acids by microbes in the hindgut. The low signals of hindgut essential amino acids seen in figures 1 and 2 may be explained by the rapid use of these compounds in protein synthesis by the microbes that are synthesizing them. The apparent lack of synchronization between cellulolysis and essential amino acid production in the hindgut is also unexpected (table 1; electronic supplementary material, figure S5). We hypothesize that the relatively low signal intensities for essential amino acids recovered in the hindgut (figure 1) may prevent accurate resolution of their relationship to ^13^C-cellulose hydrolysis using NMDS (electronic supplementary material, figure S5), leading to this anomalous result.

In contrast to the case of essential amino acids, most signals from non-essential amino acids (except Asn and Tyr) were grouped with the metabolites of cellulose catabolism in the midgut and hindgut in NMDS analyses, suggesting both the termites and hindgut microbiota probably contribute to the direct (in terms of time) production of non-essential amino acids using ^13^C-cellulose-derived carbon. Most non-essential amino acids observed in the midgut were probably derived from intermediates of glycolysis and the TCA cycle of the host termites [37], though a small contribution by residential bacteria and/or digested microorganisms cannot be ruled out. The apparent non-synchronous production of Tyr with cellulose hydrolysis may be because the synthesis of this amino acid was affected by the low availability of its precursor Phe, an essential amino acid. Amino acids associated with the serine family (i.e. Ser and Gly) and the pyruvate family (Ala) were probably derived from intermediates of the glycolytic pathway [37]. On the other hand, microorganisms assimilate ammonia in order to undertake biosynthesis of Glu and Gln. This pathway is called the GOGAT (GS/GOGAT) cycle, where OGA and ammonia are converted by glutamine synthase and glutamate synthase into Glu, from which approximately 88% of cellular nitrogen is derived and used in a variety of biosynthetic reactions [67]. This pathway is commonly found in bacteria and is also reported in some insects [68,69]. Non-synchronized signalling of OGA may indicate that the production and consumption of this substrate were affected by availability of ammonium, but syntheses of other non-essential amino acids might have been more strongly affected by the availability of residual carbon.

## 5. Conclusion

To the best of our knowledge, this study is the first metabolomic investigation of digestive processes in an arthropod, using the dampwood-feeding lower termite *H. sjostedti* as model. The analysis of ^13^C metabolites provides insights into the process of cellulose digestion and nitrogen metabolism, and is suitable for wider applications to other termite and insect species. This study has unveiled both a sequence and combination of processes converting cellulose to energy and essential anabolic nutrients, and proposes a model of efficient bioconversion that may be relevant for the industrial production of biofuels [3].

## Supplementary Material

Supporting figures

## Supplementary Material

Tables
