# Supplementary material for: Metabolomic profiling of 13C-labelled cellulose digestion in a lower termite: insights into gut symbiont function
Source: Proc Biol Sci. 2014 Aug 22;281(1789):20140990. doi: 10.1098/rspb.2014.0990 (PMC4100516; doi:10.1098/rspb.2014.0990)
Supplement: Supporting figures [file rspb20140990supp1.pdf]

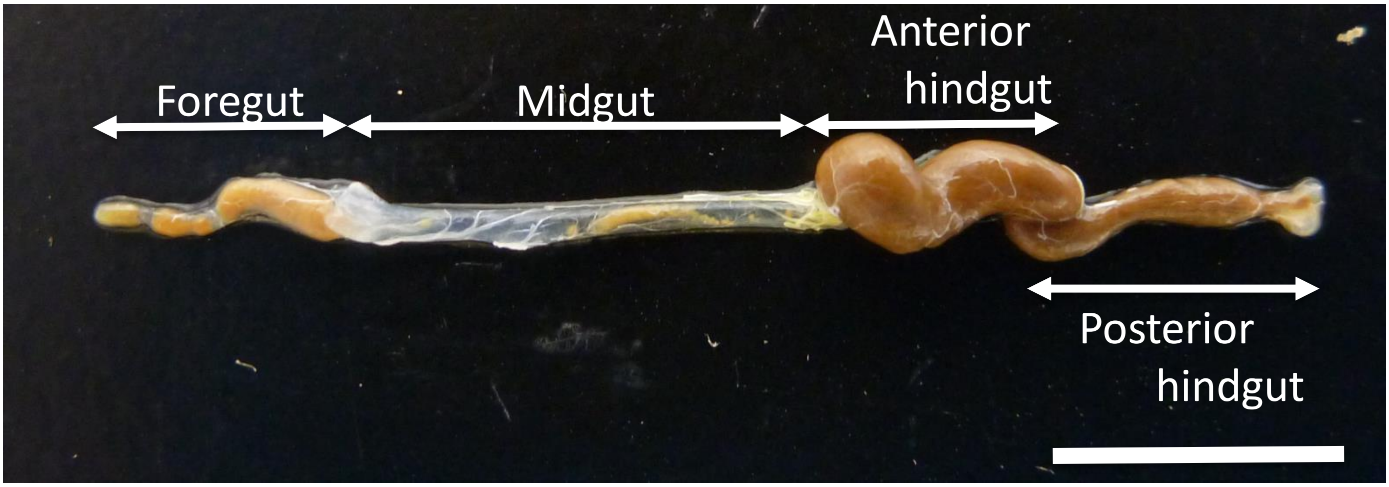

Figure S1. The gut anatomy of *H. sjostedti*. It was divided into the foregut, the midgut, and the anterior and the posterior hindgut. Bar represents 5 mm.

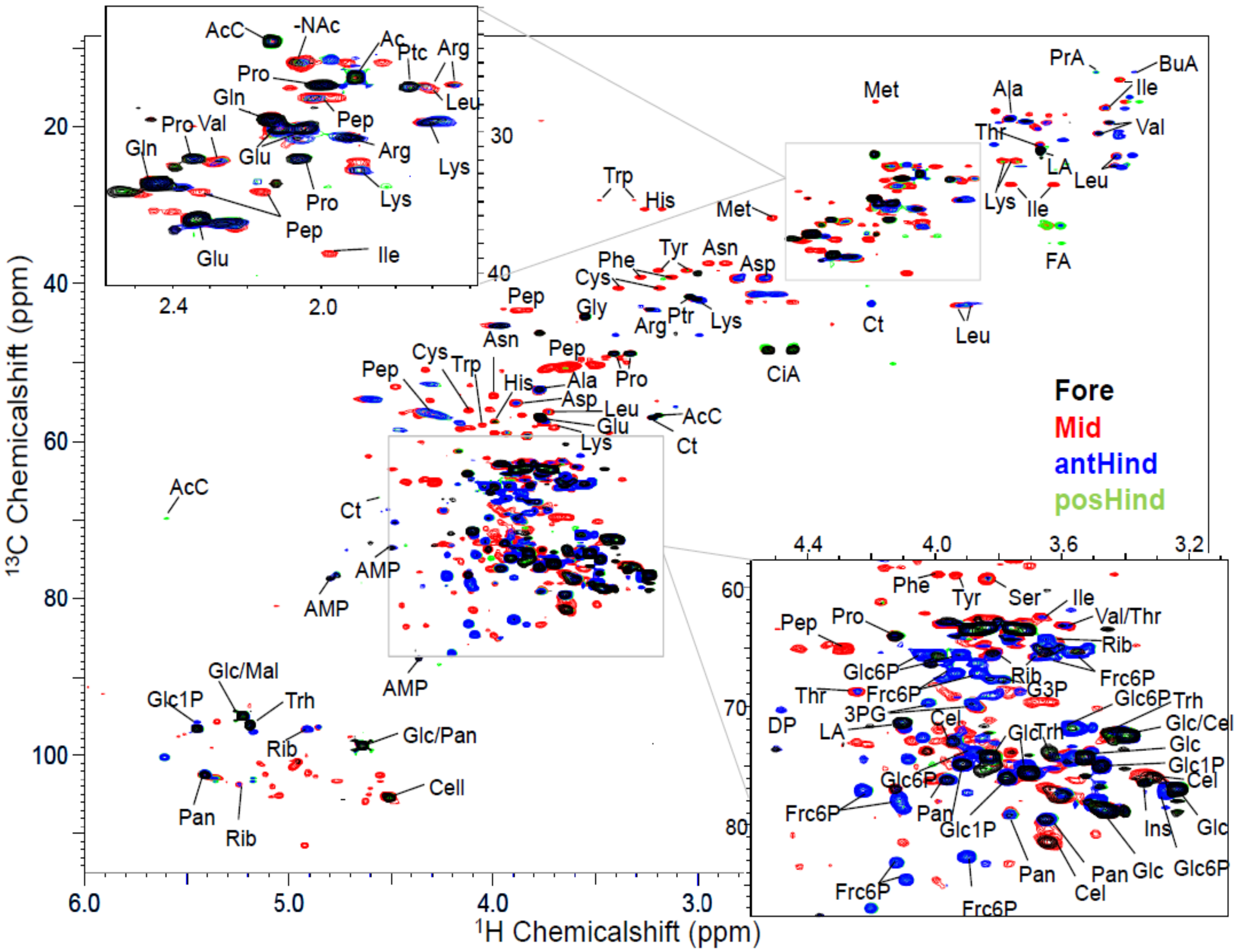

Figure S2. Overlay of ^{13}C-HSQC-NMR spectra of ^{13}C-labeled termite digestive organs (Foregut: black, Midgut: red, Anterior hindgut: blue, Posterior hindgut: green). Metabolite annotations are highlighted next to the corresponding contour signals according to Supplementary table S1.

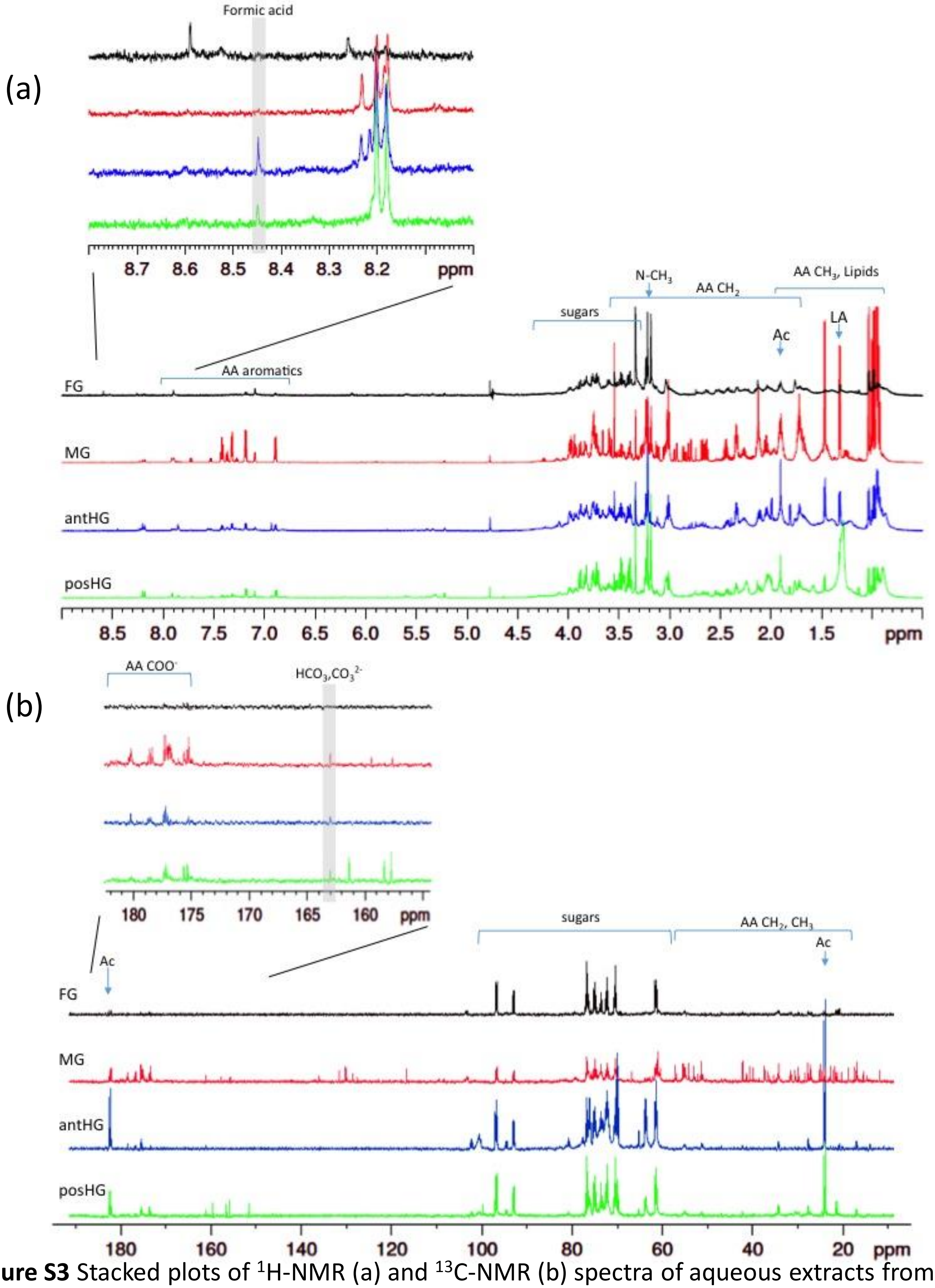

Figure S3 Stacked plots of ^{1}H-NMR (a) and ^{13}C-NMR (b) spectra of aqueous extracts from termite digestive organs (Foregut: black, Midgut: red, Anterior hindgut: blue, Posterior hindgut: green). Abbreviations are AA: amino acid, Ac: acetic acid, LA: lactic acid, respectively.

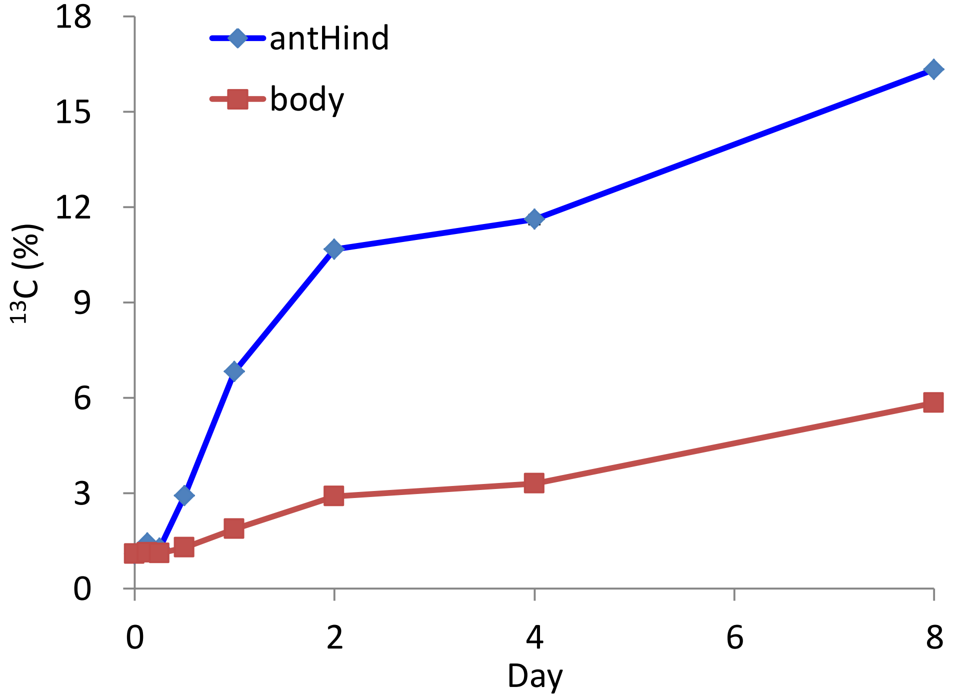

Figure S4. Temporal changes of stable isotope ratio (%) of ^{13}C in the anterior hindgut (antHind) and the body (without the salivary glands, the gut, and the head) during the experimental period, determined by IR-MS. The results indicate that ^{13}C was steadily incorporated into the gut metabolites and tissues.

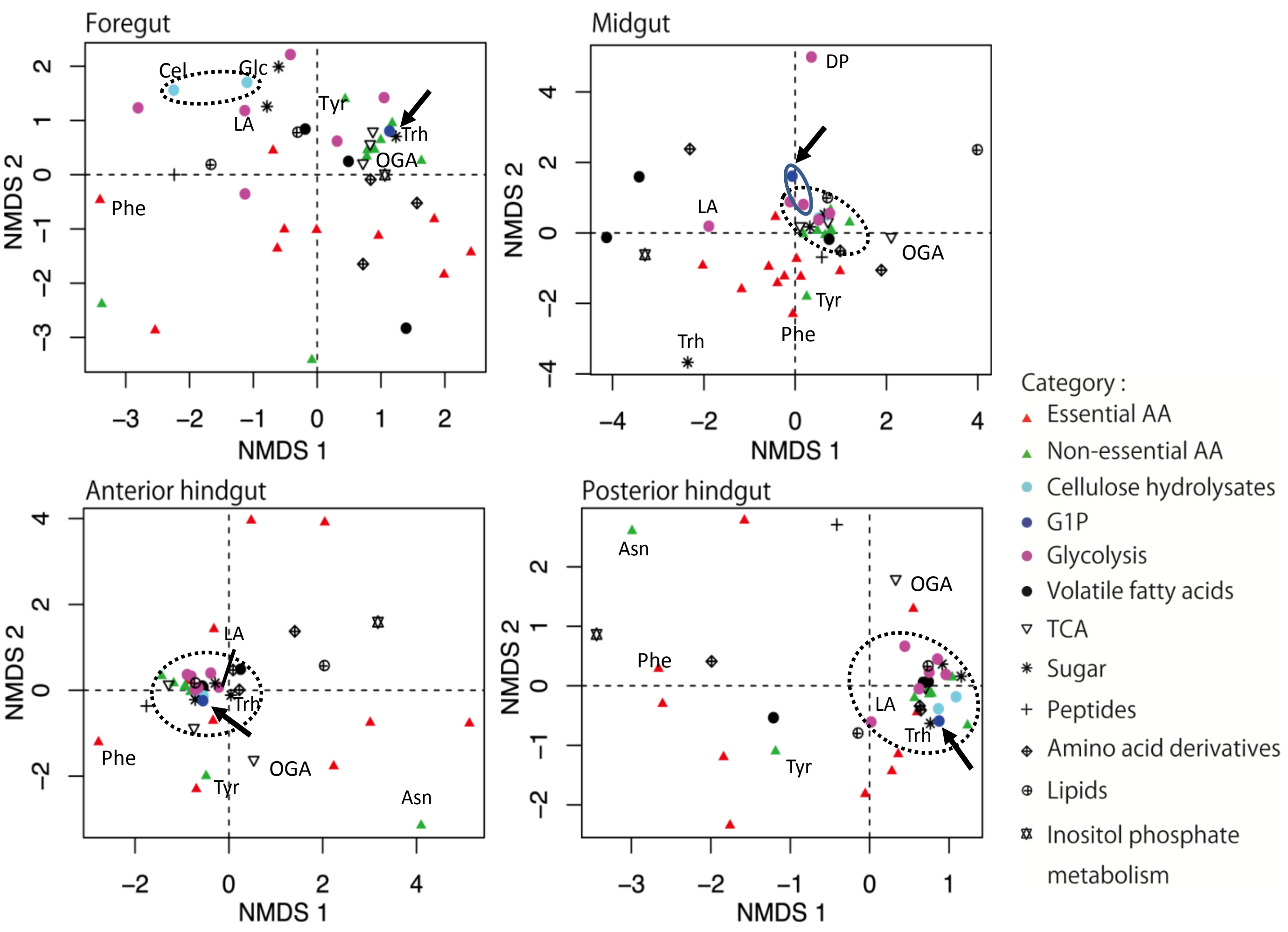

Figure S5. Ordination by NMDS of the digestive metabolites of cellulose in two-dimensional space, based on dissimilarity of temporal changes of averaged signal intensities. Groups of metabolites displaying behaviors related to cellulose hydrolysates in each gut region (dotted black circles) were inferred from a hierarchical cluster analysis with an average linkage based on Euclidean distance. Glc1P was grouped with Glc6P (blue solid circle) and the biosynthesis was not synchronized with cellulose hydrolysis. Significance of each group was assessed by MRPP ($p < 0.01$). Arrow indicates the position of Glc1P. Lactate and trehalose were grouped with cellulose hydrolysates only in the hindgut. Abbreviations are according to Supplementary table S1.
