# Supplementary material for: Metabolomic profiling of 13C-labelled cellulose digestion in a lower termite: insights into gut symbiont function
Source: Proc Biol Sci. 2014 Aug 22;281(1789):20140990. doi: 10.1098/rspb.2014.0990 (PMC4100516; doi:10.1098/rspb.2014.0990)
Supplement: Tables [file rspb20140990supp2.docx]

| Supplementary table S1. Metabolites detected in the gut of *H. sjostedti* | | | | |  |  |
| --- | --- | --- | --- | --- | --- | --- |
|  | Name (abbreviation) |  | ^1^H | ^13^C | *p*-value | Category |
| 1 | L-Methionine (Met) | -SCH3 | 2.15 | 16.7 | 2.70E-01 | Amino acids (essential) |
|  |  | C3 | 2.12 | 32.5 | 5.80E-06 |  |
|  |  | C3 | 2.19 | 32.5 | 2.40E-06 |  |
|  |  | C4 | 2.63 | 31.4 | 5.90E-09 |  |
|  |  | C2 | 3.85 | 56.6 | 1.50E-31 |  |
| 2 | L-Phenylalanine (Phe) | C3 | 3.12 | 39.1 | 1.40E-04 | Amino acids (essential) |
|  |  | C3 | 3.27 | 39.1 | 8.30E-01 |  |
|  |  | C2 | 3.98 | 58.8 | 7.10E-07 |  |
|  |  | 2,6-CH | 7.37 | 130.6 | 6.00E-33 |  |
|  |  | 4-CH | 7.39 | 130.4 | 6.30E-01 |  |
|  |  | 3,5-CH | 7.42 | 132.1 | 8.30E-01 |  |
| 3 | L-Lysine (Lys) | C4 | 1.43 | 24.1 | 3.00E-11 | Amino acids (essential) |
|  |  | C4 | 1.50 | 24.1 | 4.90E-02 |  |
|  |  | C5 | 1.72 | 29.2 | 1.00E-29 |  |
|  |  | C3 | 1.90 | 32.5 | 1.40E-20 |  |
|  |  | C6 | 3.01 | 41.8 | 3.50E-09 |  |
| 4 | L-Histidine (His) | C3 | 3.16 | 30.3 | 1.00E-60 | Amino acids (essential) |
|  |  | C3 | 3.25 | 30.3 | 9.60E-02 |  |
|  |  | C2 | 3.98 | 57.4 | 1.40E-08 |  |
|  |  | CH | 7.10 | 119.8 | 1.20E-28 |  |
| 5 | L-Tryptophan (Typ) | C3 | 3.29 | 29.2 |  | Amino acids (essential) |
|  |  | C3 | 3.47 | 29.2 | inhouse |  |
|  |  | C2 | 4.05 | 57.7 | inhouse |  |
|  |  | 7-CH | 7.52 | 114.8 | inhouse |  |
|  |  | 4-CH | 7.72 | 121.4 | 4.70E-02 |  |
| 6 | L-Isoleucine (Ile) | C5 | 0.93 | 13.9 | 5.20E-05 | Amino acids (essential) |
|  |  | C4′ | 1.00 | 17.5 | 3.60E-05 |  |
|  |  | C4 | 1.25 | 27.3 | 4.80E-05 |  |
|  |  | C4 | 1.46 | 27.3 | 1.50E-06 |  |
|  |  | C3 | 1.97 | 38.6 | 3.00E-02 |  |
|  |  | C2 | 3.66 | 62.3 | 9.60E-02 |  |
| 7 | L-Leucine (Leu) | C5,C5′ | 0.95 | 23.5 | 2.10E-08 | Amino acids (essential) |
|  |  | C5,C5′ | 0.95 | 24.9 | 1.10E-03 |  |
|  |  | C4 | 1.68 | 42.6 | 2.50E-46 |  |
|  |  | C3 | 1.72 | 42.6 | 4.80E-20 |  |
|  |  | C2 | 3.72 | 56.0 | 5.20E-05 |  |
| 8 | L-Valine (Val) | C4,C4′ | 0.98 | 19.4 | 6.20E-01 | Amino acids (essential) |
|  |  | C4,C4′ | 1.03 | 20.8 | 4.20E-03 |  |
|  |  | C3 | 2.26 | 32.0 | 1.30E-15 |  |
|  |  | C2 | 3.59 | 63.2 | 7.50E-04 |  |
| 9 | L-Threonine (Thr) | C4 | 1.31 | 22.1 | 3.00E-01 | Amino acids (essential) |
|  |  | C3 | 4.24 | 68.6 | 7.40E-02 |  |
| 10 | L-Arginine (Arg) | C3 | 1.47 | 18.9 | 2.00E-03 | Amino acids (essential) |
|  |  | C2 | 3.77 | 53.3 | 5.00E-06 |  |
| 11 | L-Alanine (Ala) | C4 | 1.64 | 26.5 | 2.10E-02 | Amino acids (nonessential) |
|  |  | C3 | 1.91 | 30.3 | 3.30E-03 |  |
|  |  | C5 | 3.23 | 43.2 | 7.70E-02 |  |
| 12 | L-Asparagine (Asn) | C3 | 2.86 | 37.2 | 3.30E-06 | Amino acids (nonessential) |
|  |  | C3 | 2.93 | 37.2 | 2.90E-01 |  |
|  |  | C2 | 3.99 | 54.1 | 2.50E-08 |  |
| 13 | L-Aspartate (Asp) | C3 | 2.68 | 39.4 | 3.90E-09 | Amino acids (nonessential) |
|  |  | C3 | 2.80 | 39.4 | 8.10E-11 |  |
|  |  | C2 | 3.88 | 55.0 | 3.80E-02 |  |
| 14 | L-Glutamine (Gln) | C3 | 2.13 | 29.0 | 5.40E-02 | Amino acids (nonessential) |
|  |  | C4 | 2.43 | 33.6 | 3.20E-09 |  |
|  |  | C2 | 3.77 | 56.9 | 2.00E-05 |  |
| 15 | L-Glutamate (Glu) | C3 | 2.06 | 29.8 | 1.80E-04 | Amino acids (nonessential) |
|  |  | C3 | 2.11 | 29.8 | 1.80E-11 |  |
|  |  | C4 | 2.34 | 36.1 | 1.70E-08 |  |
|  |  | C2 | 3.75 | 57.1 | 2.30E-03 |  |
| 16 | L-Glycine (Gly) | CH2 | 3.54 | 44.3 | 8.40E-07 | Amino acids (nonessential) |
| 17 | L-Proline (Pro) | C4 | 2.00 | 26.4 | 1.60E-06 | Amino acids (nonessential) |
|  |  | C3 | 2.05 | 31.8 | 1.20E-08 |  |
|  |  | C3 | 2.34 | 31.8 | 3.80E-08 |  |
|  |  | C5 | 3.33 | 48.9 | 3.40E-08 |  |
|  |  | C5 | 3.40 | 48.9 | 8.90E-11 |  |
|  |  | C2 | 4.12 | 64.0 | 1.20E-12 |  |
| 18 | L-Serine (Ser) | C2 | 3.83 | 59.1 | 1.40E-02 | Amino acids (nonessential) |
|  |  | C3 | 3.97 | 62.9 | 1.10E-16 |  |
| 19 | L-Tyrosine (Tyr) | C3 | 3.04 | 38.3 | 1.80E-05 | Amino acids (nonessential) |
|  |  | C3 | 3.19 | 38.3 | 7.40E-05 |  |
|  |  | C2 | 3.93 | 58.8 | 5.80E-03 |  |
|  |  | 3,5-CH | 6.88 | 118.7 | 6.00E-05 |  |
|  |  | 2,6-CH | 7.18 | 133.7 | 3.30E-13 |  |
| 20 | Cystine (CySS) | C3 | 3.19 | 80.5 | HMDB | Amino acids (nonessential) |
|  |  | C3 | 3.39 | 80.5 | HMDB |  |
|  |  | C2 | 4.11 | 56.0 | HMDB |  |
| 21 | Peptide(Pep) | AlaAla | 1.35 | 60.0 | 1.50E-20 | Precursor of peptidoglycan cell walls |
|  |  | AlaAla | 1.54 | 59.2 | 5.50E-01 |  |
|  |  | GlyPro | 2.01 | 27.4 | HMDB | Peptide |
|  |  | GlyPro | 2.06 | 24.9 | HMDB |  |
|  |  | GlyPro | 2.33 | 34.3 | HMDB |  |
|  |  | GlyPro | 3.60 | 50.4 | HMDB |  |
|  |  | GlyPro | 3.64 | 50.5 | HMDB |  |
|  |  | GlyPro | 3.71 | 50.4 | HMDB |  |
|  |  | GlyPro | 3.84 | 43.2 | HMDB |  |
|  |  | GlyPro | 4.29 | 65.1 | HMDB |  |
| 22 | Citrate (CiA) |  | 2.54 | 48.2 | 1.50E-12 | TCA cycle/fermentation |
|  |  |  | 2.66 | 48.2 | 2.80E-61 |  |
| 23 | 2-Oxoglutarate (OGA) |  | 3.00 | 38.6 | 1.70E-09 | TCA cycle/GOGAT cycle |
|  |  |  | 2.43 | 33.6 | 7.00E-58 |  |
| 24 | Succinate (SuA) |  | 2.41 | 36.9 | 3.10E-08 | TCA cycle/hydrogenosome metabolism |
| 25 | Malate (MA) | C2 | 4.29 | 73.0 | 1.60E-01 | TCA cycle/hydrogenosome metabolism |
| 26 | Acetate (Ac) |  | 1.91 | 25.9 | 2.60E-11 | Volatile fatty acid/hydrogenosome metabolism |
| 27 | Butyrate (BuA) | C4 | 0.89 | 15.9 | 7.00E-08 | Volatile fatty acid |
|  |  | C3 | 1.55 | 22.0 | 8.40E-08 |  |
|  |  | C2 | 2.15 | 42.2 | 1.50E-10 |  |
| 28 | L-Lactate (LA) | CH3 | 1.32 | 22.9 | 5.40E-17 | Glycolytic pathway |
|  |  | CH | 4.10 | 71.1 | 1.90E-09 |  |
| 29 | Propionate (PrA) |  | 1.04 | 13.0 | 3.00E-08 | Volatile fatty acid |
|  |  |  | 2.17 | 33.5 | 2.00E-05 |  |
| 30 | Putrescine (Ptc) |  | 1.76 | 26.6 | 9.70E-08 | Derivative of amino acids (polyamine) |
| 31 | Cellobiose/cellooligosaccharides (Cel) |  | 3.31 | 3.31 | 1.30E-20 | Cellulose hydrolysate |
|  |  |  | 3.53 | 3.53 | 1.40E-65 |  |
|  |  |  | 3.57 | 3.57 | 1.20E-33 |  |
|  |  |  | 3.65 | 3.65 | 1.50E-20 |  |
|  |  |  | 3.81 | 3.81 | 7.90E-28 |  |
|  |  |  | 3.88 | 3.88 | 6.80E-34 |  |
|  |  |  | 3.94 | 3.94 | 8.70E-01 |  |
|  |  |  | 4.52 | 4.52 | 5.50E-22 |  |
| 32 | Glucose (Glc) |  | 3.22 | 77.0 | 1.80E-25 | Cellulose hydrolysate (Hexose) |
|  |  |  | 3.38 | 72.4 | 5.30E-28 |  |
|  |  |  | 3.46 | 78.7 | 3.40E-05 |  |
|  |  |  | 3.53 | 74.0 | 3.10E-10 |  |
|  |  |  | 3.71 | 75.5 | 1.50E-01 |  |
|  |  |  | 3.71 | 63.5 | 6.50E-03 |  |
|  |  |  | 3.76 | 63.3 | 4.60E-01 |  |
|  |  |  | 3.83 | 74.1 | 8.50E-05 |  |
|  |  |  | 3.83 | 63.2 | 3.70E-03 |  |
|  |  |  | 3.89 | 63.4 | 9.20E-02 |  |
|  |  |  | 4.64 | 98.7 | 1.30E-02 |  |
|  |  |  | 5.23 | 94.9 | 4.30E-05 |  |
| 33 | Panose (Pan) |  | 3.28 | 77.0 | 8.00E-25 | Sugar (trisaccharide) |
|  |  |  | 3.65 | 79.4 | 1.70E-61 |  |
|  |  |  | 3.76 | 78.9 | 5.80E-06 |  |
|  |  |  | 3.96 | 76.0 | 1.80E-02 |  |
|  |  |  | 5.41 | 102.2 | 1.10E-06 |  |
|  |  |  | 3.59 | 77.1 | 8.50E-12 |  |
|  |  |  | 3.60 | 77.7 | 6.20E-87 |  |
| 34 | Ribose (Rib) |  | 3.87 | 69.9 | 2.70E-03 | Sugar (pentose) |
|  |  |  | 4.10 | 71.8 | 7.50E-02 |  |
|  |  |  | 3.66 | 64.1 | 1.50E-18 |  |
|  |  |  | 3.66 | 65.7 | 3.10E-21 |  |
|  |  |  | 3.77 | 64.1 | 2.80E-34 |  |
|  |  |  | 3.80 | 73.0 | 1.90E-14 |  |
|  |  |  | 3.83 | 65.6 | 1.10E-07 |  |
|  |  |  | 3.99 | 85.3 | 2.50E-35 |  |
|  |  |  | 4.91 | 96.5 | 1.10E-07 |  |
| 35 | Trehalose (Trh) |  | 3.45 | 72.5 | 1.40E-16 | Sugar (disaccharide) |
|  |  |  | 3.64 | 73.8 | 1.00E+00 |  |
|  |  |  | 3.81 | 75.0 | 8.80E-27 |  |
|  |  |  | 3.85 | 75.4 | 7.50E-79 |  |
|  |  |  | 5.20 | 95.9 | 2.30E-08 |  |
| 36 | 2-Phospho-glycerate (2PG) |  | 3.68 | 64.8 | 2.00E-02 | Glycolytic pathway |
| 37 | 3-Phospho-glycerate (3PG) |  | 3.89 | 69.5 | 8.30E-15 | Glycolytic pathway |
|  |  |  | 4.02 | 69.5 | 2.60E-25 |  |
|  |  |  | 4.15 | 75.4 | 4.20E-63 |  |
| 38 | Fructose-6-phosphate (F6P) |  | 3.57 | 65.3 | 2.00E-27 | Glycolytic pathway/pentose phosphate pathway |
|  |  |  | 3.86 | 67.0 | 6.40E-02 |  |
|  |  |  | 3.90 | 82.5 | 3.00E-01 |  |
|  |  |  | 3.94 | 67.0 | 7.10E-01 |  |
|  |  |  | 4.10 | 84.4 | 4.60E-08 |  |
|  |  |  | 4.12 | 77.7 | 6.60E-30 |  |
|  |  |  | 4.13 | 83.0 | 6.40E-02 |  |
|  |  |  | 4.23 | 76.9 | 1.20E-06 |  |
| 39 | Glucose-1-phosphate (G1P) |  | 3.47 | 74.9 | 1.40E-04 | Cellulose hydrolysate/glycogenolysis |
|  |  |  | 3.77 | 75.8 | 2.60E-02 |  |
|  |  |  | 3.92 | 74.7 | 1.70E-09 |  |
|  |  |  | 5.45 | 96.5 | 1.40E-02 |  |
| 40 | Glucose-6-phosphate(G6P) |  | 3.51 | 77.9 | 4.20E-57 | Glycolytic pathway/pentose phosphate pathway |
|  |  |  | 3.57 | 71.7 | 5.70E-21 |  |
|  |  |  | 3.71 | 75.0 | 5.10E-04 |  |
|  |  |  | 3.88 | 73.7 | 1.70E-18 |  |
|  |  |  | 3.92 | 65.5 | 9.80E-22 |  |
|  |  |  | 4.00 | 65.6 | 1.40E-04 |  |
| 41 | Dihydroxyacetone phosphate (DP) |  | 3.79 | 68.6 | 2.00E-07 | Glycolytic pathway |
|  |  |  | 4.49 | 70.1 | 5.00E-13 |  |
| 42 | Glycerol-3-phosphate (G3P) |  | 3.78 | 67.5 | 1.50E-25 | Glycerolipid metabolism |
|  |  |  | 3.82 | 67.5 | 5.20E-12 |  |
| 43 | Acetylcarnitine (AcC) |  | 2.14 | 23.2 | 2.30E-15 | Derivative of amino acids |
|  |  |  | 2.49 | 43.0 | 1.90E-15 |  |
|  |  |  | 2.63 | 43.1 | 4.50E-16 |  |
|  |  |  | 3.60 | 71.0 | 3.20E-12 |  |
|  |  |  | 3.85 | 70.9 | 1.80E-19 |  |
|  |  |  | 5.60 | 69.7 | 1.20E-14 |  |
|  |  | NCH3 | 3.19 | 56.5 | 4.10E-09 |  |
| 44 | Carnitine (Ct) | NCH3 | 3.23 | 56.9 | 8.70E-31 | Derivative of amino acids |
|  |  |  | 2.43 | 45.7 | 1.20E-07 |  |
|  |  |  | 3.42 | 73.0 | 5.40E-17 |  |
|  |  |  | 4.55 | 66.9 | 1.60E-02 |  |
| 45 | Fatty acid (FA) |  | 0.89 | 16.8 | 8.70E-25 | Linoleic acid metabolism |
|  |  |  | 1.27 | 24.7 | 6.40E-87 |  |
|  |  |  | 1.29 | 35.5 | 9.30E-16 |  |
|  |  |  | 1.29 | 32.1 | 4.60E-12 |  |
|  |  |  | 1.57 | 27.6 | 9.10E-51 |  |
|  |  |  | 2.01 | 30.0 | 8.00E-25 |  |
| 46 | Scyllo-Inositol (Ins) |  | 3.34 | 76.3 | BMRB | Inositol phosphate metabolism |
